# Universal HIV testing and treatment and HIV stigma reduction: a comparative thematic analysis of qualitative data from the HPTN 071 (PopART) trial in South Africa and Zambia

**DOI:** 10.1111/1467-9566.13208

**Published:** 2020-10-21

**Authors:** Lario Viljoen, Virginia A. Bond, Lindsey J. Reynolds, Constance Mubekapi‐Musadaidzwa, Dzunisani Baloyi, Rhoda Ndubani, Anne Stangl, Janet Seeley, Triantafyllos Pliakas, Peter Bock, Sarah Fidler, Richard Hayes, Helen Ayles, James R. Hargreaves, Graeme Hoddinott, the HPTN 071 (PopART) study team

**Affiliations:** ^1^ Department of Paediatrics and Child Health Faculty of Medicine and Health Sciences Desmond Tutu TB Centre Stellenbosch University Stellenbosch South Africa; ^2^ Department of Sociology and Social Anthropology Stellenbosch University Stellenbosch South Africa; ^3^ Zambart School of Public Health Ridgeway Campus University of Zambia Lusaka Zambia; ^4^ Global Health and Development Department Faculty of Public Health and Policy London School of Hygiene and Tropical Medicine London UK; ^5^ International Center for Research on Women Washington DC USA; ^6^ Department of Public Health, Environments and Society Faculty of Public Health and Policy London School of Hygiene and Tropical Medicine London UK; ^7^ Imperial College NIHR BRC Imperial College London London UK; ^8^ Department of Infectious Disease Epidemiology, Faculty of Epidemiology and Public Health London School of Hygiene and Tropical Medicine London UK

**Keywords:** universal test and treat, sub‐Saharan Africa, stigma, responsibilisation, normalisation

## Abstract

Despite continued development of effective HIV treatment, expanded access to care and advances in prevention modalities, HIV‐related stigma persists. We examine how, in the context of a universal HIV‐testing and treatment trial in South Africa and Zambia, increased availability of HIV services influenced conceptualisations of HIV. Using qualitative data, we explore people’s stigma‐related experiences of living in ‘intervention’ and ‘control’ study communities. We conducted exploratory data analysis from a qualitative cohort of 150 households in 13 study communities, collected between 2016 and 2018. We found that increased availability of HIV‐testing services influenced conceptualisations of HIV as normative (non‐exceptional) and the visibility of people living with HIV (PLHIV) in household and community spaces impacted opportunities for stigma. There was a shift in community narratives towards individual responsibility to take up (assumingly) widely available service – for PLHIV to take care of their own health and to prevent onward transmission. Based on empirical data, we show that, despite a growing acceptance of HIV‐related testing services, anticipated stigma persists through the mechanism of shifting responsibilisation. To mitigate the responsibilisation of PLHIV, heath implementers need to adapt anti‐stigma messaging and especially focus on anticipated stigma.

## Introduction

Over half of the 37.9 million people living with HIV worldwide are in southern and eastern Africa (UNAIDS 2019). The past 30 years have seen improvements in effective treatment (Boswell *et al*. 2018), expanded access to HIV care (WHO 2015) and advances in prevention modalities, including HIV treatment as a form of prevention (Cohen *et al*. 2016). However, HIV‐related stigma, one aspect of the social landscape of HIV, persists (Maughan‐Brown *et al*. 2018).

Stigma is linked to people’s anxieties, including the fear of disease and death, and moral concerns about sex or sexuality (Shefer *et al*. 2002). Physical (symptoms/side effects) and social (sex, morality, identity) markers of HIV are highly stigmatised (Persson 2013). People affected by HIV may be part of marginalised communities (WHO 2017) who are blamed, or held responsible, for their diagnosis and their perceived moral transgressions (Shen *et al*. 2019). Historically, the progression of HIV infection made an HIV diagnosis difficult to hide due to physical symptoms. As antiretroviral therapy (ART) became available, the side effects of treatment became stigmatised markers (Persson 2013). New treatment regimens have fewer side effects, but many PLHIV are fearful of being seen accessing HIV treatment, or being identified as living with HIV (Bond *et al*. 2019). As treatment becomes widely available, and ART is posited as an HIV prevention modality, PLHIV are increasingly held responsible for accessing care and preventing onward transmission (Carrasco *et al*. 2017).

HIV ‘stigma’ is sometimes misconstrued as a proxy for all social experiences of disease (Bonnington *et al*. 2017) and may be changing alongside the evolving HIV treatment landscape (Mall *et al*. 2013). As more HIV services become available, researchers have theorised that stigma in communities may either decrease (Squire 2010), or increase (Chan and Tsai 2016, Roura *et al*. 2009a). Others have argued that even in an era of ‘universal’ testing and treatment (UTT) (Chan *et al*. 2015), stigma continues to contribute to morbidity (Bonnington *et al*. 2017) and creates service access inequalities (Hatzenbuehler *et al*. 2013). There is limited information on how communities respond to increasingly available HIV services and the impact on stigma.

HIV stigma has been associated with how people access HIV health services, including delaying diagnosis (Treves‐Kagan *et al*. 2016) and treatment initiation (Sayles *et al*. 2009), inhibiting use of prevention tools (UNAIDS 2017), limiting status disclosure (Katz *et al*. 2013), complicating adherence (Rueda *et al*. 2016) and poorer health outcomes overall (Peitzmeier *et al*. 2015).

Researchers, policy makers and activists present the ‘normalisation’ of HIV, or the re‐conceptualising of the infection as a chronic condition, as a way to counter the othering of PLHIV (Moyer and Hardon 2014, Roura *et al*. 2009a). By contrast, fears of involuntary disclosure and being marked as living with HIV (‘being visible’), and being held responsible for HIV acquisition, treatment and transmission are part of evolving stigma dynamics (Beckmann 2013).

We analysed people’s experiences of HIV‐related stigma in the context of a community‐randomised controlled HIV prevention trial, HPTN 071 (PopART) (Hayes *et al*. 2014) in Zambia and South Africa (Hayes *et al*. 2014). The interventions included door‐to‐door HIV testing and treatment for PLHIV regardless of CD4 count. The trial was the largest study investigating the impact of UTT, prior to World Health Organisation (WHO) changes in guidelines promoting HIV treatment for all PLHIV. Community HIV care providers (CHiPs) provided the in‐home intervention. Being visited at home through the trial intervention introduced new routes to service access that intersected with social processes of stigma. The trial showed a significant reduction in population level HIV incidence amongst communities receiving the household testing intervention. However, quantitative analyses of the effect of the study intervention showed no impact on HIV stigma levels (Stangl *et al*. 2020). We used qualitative data to explore study community members’ stigma‐related experiences. Specifically, we examined how increased availability of HIV services in intervention communities influenced conceptualisations of HIV as normative (or no longer exceptional), how the visibility of PLHIV in household and community spaces impacted stigma, and how greater opportunity for service access influenced narratives of PLHIV’s responsibility for their own health.

## Theoretical background

Link and Phelan (2001: 377) defined stigma as ‘elements of labelling, stereotyping, separation, status loss and discrimination’ that co‐occur with ‘enabling power situations’. Maluwa *et al*. (2001: 6) noted that stigma is not a naturally occurring (or static) phenomenon, but rather a process of devaluation created in social contexts by individuals and communities. We employ the concepts of normalisation, visibility and responsibilisation to understand how stigma is either amplified or mitigated in the context of increased access to HIV services, specifically in Zambia and South Africa.

### Normalisation and exceptionalism

At a global scale, HIV/AIDS was historically treated as an exceptional condition in need of an exceptional response (Hardon and Moyer 2014), due to medical uncertainty surrounding the virus, the physical deterioration of affected patients, and the lack of effective treatment (de Cock and Johnson 1998). However, in the late 1980s, there were calls from researchers in the global north for a shift in focus towards quality of life and long‐term survival of patients by managing HIV as a chronic condition (McGrath *et al*. 2014). Notionally, chronic conditions that can be treated, managed and controlled can be ‘normalised’, with patients accepted and ‘re‐integrated into productive and social life’ (Roura *et al*. 2009b: 2) and as ‘self‐regulating, healthy citizens’ (Squire 2010: 408).

In order to position HIV as ‘normal’, health specialists across contexts promoted the implementation of HIV testing as part of routine care (Johnson 2019, Wise 2008). Early on in the epidemic and in the absence of effective HIV treatment, health providers emphasised the importance of (exceptional) lengthy and detailed pre‐test counselling for groups that were considered to be ‘high risk’ (Bassett *et al*. 2007, Johnson 2019). Over time, routine HIV testing was increasingly introduced as part of government programmes to all adults (including in Zambia in Africa) in order to improve case findings, and potentially, to reduce stigma, and to position the act as non‐exceptional (Mall *et al*. 2013).

However, it is the roll‐out of ART programmes across contexts that was expected to further the process of normalisation (Persson 2013). For instance, Squire (2010:407) noted that, in the UK, as more people became eligible for treatment, taking on the role of a ‘regular, unremarkable citizens’ would be increasingly possible. McGrath *et al*. (2014:305), however, noted that ‘the transition [of HIV] to a manageable chronic disease has not unfolded at the same rate or in the same way in all populations’. The shift of HIV to a chronic condition is linked to stable access to HIV care, and was, at least historically, therefore more difficult to achieve in Low and Middle Income Countries (LMIC) and/or marginal populations where structural and social constraints undermine access to services.

As treatment became more readily available in LMIC, activists also anticipated that through the re‐conceptualising of HIV as a manageable condition, stigma would decrease and disclosure and HIV testing could increase (Roura *et al*. 2009a). This echoes sentiments from research in the UK where normalisation is presented as a means to counter HIV stigma by positioning the illness as a ‘regularised part of biological and social life’ (Squire 2010: 410).

However, PLHIV and communities affected by HIV have continued to be positioned as outside of the injunctive norm and treated as exceptional. The causal link between more effective biomedical responses and social normalisation of illness remains unclear (Moyer and Hardon 2014). From research in Australia, Persson (2013) questions whether bodies that have historically been positioned as dangerous and contagious can be reimagined as non‐infectious and harmless through the introduction of biomedical interventions, such as treatment as prevention. The same questions persist in southern Africa, where African bodies have repeatedly been presented as either hypersexualised or problematic (Reid and Walker 2005). The implementation of UTT is dependent on the roll‐out and uptake of HIV testing and treatment at scale. Little is known about the extent to which these increased services will become part of ‘normalised’ community narratives.

### Visibility

Historically, both the literal and metaphorical ‘visibility’ of HIV has shaped social responses to the epidemic. Goffman (1963) categorised stigmatising conditions in terms of either ‘discredited’ or ‘discreditable’ identities. Conditions that are linked to discredited identities are visible through stigmatising markers, whereby the ‘inferior status’ of the person cannot be hidden. As the stigmatising condition is public, the affected person is tasked with developing coping strategies for when they experience discrimination. Brouwer (1998: 1233) in his research on HIV/AIDS and visibility in the USA, for instance, explained that affected persons have to confront the challenge of ‘assuaging the discomfort that [a] visible stigma compels in others’. Discreditable identities, alternatively, are conditions that can be hidden from others and persons affected must manage ‘when, how, and to whom’ hidden conditions are disclosed. Similarly, Steward *et al*. (2008) from HIV research in India noted that, persons with discreditable conditions may try to maintain the boundaries, keeping their discreditable status from becoming a discredited identity.

Early on in the epidemic, the absence of any treatment meant that HIV was especially physically visible to others (Sitas and Newton 2000) and patients were easily identifiable (Persson 2013). As combination therapy became available across the globe, it became increasingly rare for PLHIV to present with the physical symptoms reflecting advanced stages of AIDS, positioning the condition, potentially, as no longer discrediting, but rather as discreditable.

However, initiating HIV treatment meant that some PLHIV became visible in other, often unexpected ways. Many PLHIV experienced visible symptoms related to the side effects of treatment, including lipodystrophy (visible redistribution of body fat). In addition, when people took the (often large number of) tablets into their homes, the medication acted as a potential visible indicator of living with the illness, especially in households in African contexts where privacy was not always possible (Horter *et al*. 2017). Across contexts, many PLHIV continue to hide their medication for fear of involuntary disclosure. In respective studies, Kalichman *et al*. (2019a) and Mackworth‐Young *et al*. (2020) for instance, noted that many PLHIV in southern Africa hide their treatment as a strategy to avoid identification and stigma. In addition, although HIV testing and treatment has become common‐place in most African contexts, people remain wary of inadvertent status disclosure and its social consequences (Pai *et al*. 2013) HIV services are still not geared towards maintaining confidentiality and clients often note their fears of ‘being seen’(Bond *et al*. 2019). However, it is not visibility itself, but rather the social implications of being associated with HIV that is the concern. Treichler (2002: 261) noted that HIV is ‘an epidemic of signification’ and that a positive diagnosis is a ‘nexus where multiple meanings, stories, and discourses intersect and overlap, reinforce, and subvert one another’. Often, these meanings are inscribed with stigmatising connotations, including associations with moral transgressions. Across the world, these notions are informed by the negative attitudes that people hold towards those that are thought to be more responsible for HIV transmission, notably, men who have sex with men, people who inject drugs, sex workers, people who are seen as ‘promiscuous’, African sexualities in general and, more recently, PLHIV who are not on treatment (Bond *et al*. 2016, Persson 2013).

### Responsibilisation

The notion of responsibilisation, derived from the Foucauldian analysis of governmentality and power, has been incorporated into understandings of HIV management, and specifically, HIV self‐management. According to Foucault (1991), ‘governmentality’ is the ways in which power or governance can be exercised through the making and maintaining of subjects and through managing responsibility‐taking of these subjects. Through subjects that are self‐governing, the state is able to shift the responsibility of governance onto citizens. In this way, responsibilised citizens are no longer obedient dependent subjects, but rather are themselves producing government ends (Foucault 1977, 1991). Authority is self‐imposed, rather than the externally enforced agent of the state (Rangel and Adam 2014).

HIV treatment was initially either unavailable or difficult to access. In South Africa, for instance, after 2004, ART was made available in the public health system (National Department of Health 2004). Through activism, treatment access was positioned not only as a human right, but as the responsibility of the state (Robins 2006). Thereafter, ART in South Africa, as in many other contexts, became available, but administered by specialised doctors. At the time, the process of initiating treatment was highly regulated and adherence closely monitored (Koenig *et al*. 2006). Patients had very little opportunity for agency or control over their treatment journey. Over time, the WHO advocated for more PLHIV to be able to access ART. To meet the demand of initiation and managing more patients on treatment programmes, treatment initiation in Africa (and beyond) shifted from the responsibility of specialised doctors to trained nurses (Crowley and Mayers 2015). In many places, including South Africa and Zambia, HIV treatment distribution moved from health facilities to off‐site adherence clubs (Campion 2015) and even in‐home delivery of treatment in some instances (Wringe *et al*. 2010). In line with global trends, PLHIV in Africa have been encouraged to engage with treatment as active citizens – autonomous care users rather than passive recipients of health services (Newman *et al*. 2015, Robins 2006).

The process of responsibilisation is posited against the theoretical notion of the ‘logic of choice’ (Mol 2008). Accordingly, the supposition is, if persons have access to HIV testing, they will do ‘the right thing’ to ‘know their status’ (Wallace *et al*. 2011). Similarly, if PLHIV are provided with sufficient information and effective treatment, they would be ‘good citizens’ who avoid transmission, access treatment and take care of their own health (Rangel and Adam 2014). In this way, complex issues are reduced to matters of choice, where ‘technologies and information are treated as neutral aids to making the right decisions’ (Beckmann 2013). People who ‘choose’ not to make the ‘logical’ decision to test, access treatment or prevent transmission are therefore blamed because of these ‘irresponsible acts’ (Bond *et al*. 2016). Researchers in the USA have noted that PLHIV are often subject to intersecting vulnerabilities – including stigma, economic and social burdens – and positioning clients as responsibilised citizens potentially compound existing inequalities (McSwiggin 2017). This is also reflected in the experience of many people in sub‐Saharan Africa affected by HIV.

### Stigma and the HIV landscape in Zambia and South Africa

As HIV prevalence in adults in Zambia (11,3%) and South Africa (20,4%) remains high (UNAIDS 2018a, 2018b), efforts to address stigma have been prioritised by many health specialists.

Since the early 2000s, ART has become increasingly available in Zambia and South Africa and HIV testing has become part of the routine public health care. Over time, and as both countries adopted changing WHO guidelines, more people became eligible for HIV treatment (Hayes *et al*. 2019). As in other contexts, increased available to ART in Zambia and South Africa was expected to aid in the normalisation of HIV (Persson 2013) and at a time when the HIV prevalence ranges from between 10‐30% in many communities in sub‐Saharan Africa (Grobler *et al*. 2017, HSRC 2018), living in a family affected by HIV is becoming descriptively normative.

With more people accessing HIV testing and treatment, aspects related to the visibility of HIV is being balanced on multiple platforms. In addition to the possibility of ART as visible marker of an HIV diagnosis in the privacy of the home (Horter *et al*. 2017, Kalichman *et al*. 2019b), in both Zambia and South Africa, the spatial organisation of health services in facilities have been identified as an avenue for signifying HIV status and as a risk for PLHIV to be recognised as living with the illness (Bond *et al*. 2019).

The notion of responsibilised citizens in the Zambian and South African context is particularly pertinent at this stage, as ART is increasingly positioned as a viable HIV prevention strategy (Hayes *et al*. 2019). The potential exists for increased pressure on PLHIV to be positioned as responsibilised citizens who know their status, adhere to treatment, and by doing so, carry the responsibility of preventing onward transmission.

We employ the constructs of normalisation, visibility and responsibilisation in Zambian and South African communities where universal access to HIV testing was rolled out prior to the adoption of the strategy on a global scale.

## Methods

### Setting

The HPTN 071 (PopART) cluster randomised three‐armed controlled trial, implemented in South Africa and Zambia from 2013 to 2018, included 21 study communities (9 in South Africa, 12 in Zambia). All 21 communities were classified as urban areas and located relatively close to district towns. Most of the sites were porous with high levels of mobility (see Co‐author 2016 and Co‐author 2018). Housing consisted of a mix of ‘informal’ and ‘formal’ housing across communities. Sites were defined around public health facility catchment areas with households located in close proximity to facilities. HIV prevalence at baseline (2013) varied across communities. In one triplet of trial communities in South Africa, HIV prevalence varied between 3% and 12% while others ranged from 19% to 35%. In Zambia, prevalence across the 12 communities ranged from 16% to 26%.

The trial intervention package included door‐to‐door HIV‐testing services and early access to ART (Hayes *et al*. 2014). A study‐specific cadre of approximately 740 CHiPs (recruited from and mostly resident in the study communities) were employed to implement the HIV prevention package in the 14 intervention communities. The 7 control communities received the standard of care and no additional testing or treatment services (Hayes *et al*. 2019).

As part of an ancillary study on stigma (Hargreaves *et al*. 2016), we included a longitudinal qualitative cohort, conducted in 13 of the study communities (see Hoddinott *et al*. 2018).

### Study design and sampling

The qualitative cohort included 150 households in 9 intervention and four control communities. Through an open sampling strategy we recruited households based on the principles of sampling for diversity (Dattalo 2010) to include participants of varying ages, genders, housing types, household composition and locations in the community. At least 50% of households included one or more PLHIV (Co‐author 2018). In South Africa, we analysed data from 74 households (with 61 PLHIV) and 60 households (with 32 PLHIV) in Zambia.

### Data collection

We collected data between 2016 and 2018 and interacted with each household ~5‐12 times^1^ over the course of 12–24 months. Field teams facilitated more than 1000 interviews and group discussions, informed by the ethnographic research principle of repeated interactions and ‘deep hanging out’ (Geertz 1998). We employed participatory methods such as household map drawings, kinship maps and timeline activities to elicit conversation. We recorded interviews and collected detailed field notes.

### Data analysis

We undertook an exploratory analysis employing a reflexive approach in order to contribute to theory‐generation. We included routine, structured written reflections by data collectors and a targeted review of all primary data. All recordings were transcribed verbatim, anonymised and translated into English.

We made use of a two‐phased coding approach. Firstly, we identified all extracts where participants, including PLHIV, referred to experiences of accessing HIV‐related services or not. During the second phase, four of the co‐authors coded these extracts thematically, informed by the analytic objectives (Braun and Clarke 2006). Themes were cross‐checked and confirmed between coders. We present the findings as case examples. All interpretations of data were discussed with the in‐country teams.

### Ethical considerations

Approval was provided by the London School of Hygiene and Tropical Medicine, University of Zambia, and Stellenbosch University research ethics committees. All participants signed written informed consent. We use pseudonyms throughout to protect participant confidentiality.

## Findings

### HIV at community level: exceptional or the new norm?

The implementation of the HPTN 071 (PopART) HIV prevention package delivered at household level introduced new forms of interaction between community members and the health system. CHiPs scheduled annual household visits with community members and offered multiple opportunities to test for HIV in home (Hayes *et al*. 2014). Many community members in both Zambia and South Africa reported that they tested more often, or whenever they encountered the CHiPs in their neighbourhood. The continued presence of the CHiPs meant that some participants described testing with the CHiPs as routine. For example, Mapalo (26), from a Zambian intervention community, described testing with the CHiPs as ordinary, and even as a way of assisting the CHiPs to ‘do their jobs’. He described how he was ‘impressed with their efforts’ and he ‘wanted to give time to the people who have made an effort to come and visit [him] at home’.

For others, testing with the CHiPs was relayed in a matter‐of‐fact way. Eva (49) lived in another Zambian intervention community. She told the story of her HIV test with the CHiPs:

They just came as you have come … I asked them to come in and they sat where you are sitting. They explained that they are going around testing people. They asked me if they could test me, and I said they could test me. And then they tested me.

Tracy (35) from a South African intervention community, described testing with the CHiPs as ordinary, and even as a convenient distraction:

Tracy: I do it [HIV test] just here in the road. I just say, I am sitting here, I am going nowhere. Come, do it just here.

Researcher: Do they just walk in here then you say; ‘It’s time?’

Tracy: No, I keep on bothering them in the road, I am very [serious] about my testing. I just call them: Hey! Come, come! I want to see what’s going on (laughing).

By contrast, HIV testing outside of the intervention communities was mostly available at health facilities, or intermittently, at mobile testing sites during targeted drives. Many participants described testing at public health facilities as either burdensome, or once‐off experiences incorporated into other health services, including antenatal care, family planning or chronic care. Thandi (35), from South Africa, jokingly recounted how provider‐initiated HIV testing was experienced as mandatory at the local health facilities. She explained: ‘there, we are tested by force (laughing). They don’t beg [keep asking] us there [like the CHiPs do]’.

Alex (48), from a Zambian control community, described the taxing experience of testing for HIV at government facilities:

“You really need to come early. You find that you just brush your teeth before eating anything, [and] rush to the clinic … Sometimes you will find few people and at times a lot, it differs … You find that you are coming out of this place [clinic] at 14, 15 or even 16 hours [two, three of four o’clock]. Sometimes [we] quit because of queuing and spending a lot of time there”.

HIV testing with the CHiPs was described as mundane when compared to the effort of accessing HIV testing at health facilities. Routine, in‐home HIV testing can be seen as a mechanism of normalisation, where testing is a way to render HIV as non‐exceptional. This could pave the way for positioning an HIV diagnosis as normalised or as a ‘regularised part of biological and social life’ (Squire 2010: 410) and as a way to counter different forms of stigma.

While the testing process was described as ordinary, a closer examination of participant responses to a potential HIV‐positive diagnosis, revealed a different narrative. Tracy, who was receptive to the intervention and even enthusiastic about testing, described how she would respond if she were to test positive:

What comes to mind? For me, I am not actually afraid, but everyone else, other people will think, geez, she has HIV! …I told myself, if I must have it, then I will throw myself under a train. I won’t still be able to live with such an illness (exhales loudly). I won’t be able to live with it, really (pause). I wouldn’t (pause). It will just have to be the end of my life (pause). I would mention it to my family and then I would tell them but (pause, reconsiders) I can’t, I am too… (pause). I would probably die quick (pause), because I stress too much (laughs).

Although Tracy does not report ever having experienced stigma related to HIV, she anticipates a severe reaction. Turan *et al*. (2017: 284) describe this as anticipated stigma or the ‘expectation of repercussions in the future; these are beliefs by PLHIV that others will treat them negatively due to their HIV status’. Eva, whom in the excerpt above described how she tested with the CHiPs who knocked on her door, relayed how when she received her positive test results: ‘I was just afraid, like, you are feeling guilty’. For Eva, the normalisation of testing did not counter her initial feelings of fear or guilt.

For Tracy, Eva and others in our cohort, the act of testing for HIV was positioned as ‘ordinary’ and acceptable, but a (potential) positive diagnosis was still viewed as life altering. The acceptability of regular household HIV testing did not necessarily translate into normalisation of HIV.

Despite the acceptance of HIV testing, the high prevalence of HIV, the daily occurrence of HIV across settings and the labelling of HIV as ‘normal’ by some local residents in both Zambia and South Africa, this did not translate into normalisation per se. An HIV‐positive diagnosis in these settings was still accompanied by life adjustments (Seeley *et al*. 2019) and by ‘social peril’ (Alonzo and Reynolds 1995) if relationships were strained and/or individuals or sub‐populations were otherwise marginalised (de Wet 2019).

In Zambia, health workers explained that HIV is ‘normal’ or ‘similar to malaria’. Brenda (25), from a Zambian community explained:

These days they have stopped [using derogatory terms] … Now it is just like Malaria. They don’t laugh at each other these days … you just have to adhere to the medication … and your body will be healthy.

While increased services meant HIV was, for some, more ‘normal’, for many participants, an HIV diagnosis was still considered a life‐altering event, associated with anticipated stigma.

### The visibility of HIV and HIV services

The CHiPs conducted more than 150,000 annual door‐to‐door HIV tests over the three‐year intervention period and numerous household follow‐up visits. The CHiPs, wearing their recognisable uniforms, served as a visible reminder of the ongoing trial with a focus on HIV prevention.

In the intervention, HIV‐testing services were provided in the private sphere of the home and participants were effectively able to avoid public health facilities. Participants frequently expressed similar fears over the perceived lack of confidentiality and concerns of being recognised when accessing HIV‐related services at public health facilities. In control communities, there were few HIV‐testing or ‐treatment options outside of public health facilities. In Zambia, Naomi mentioned PLHIV avoiding accessing HIV treatment at her local clinic: ‘they do not want others to be seeing them when going to collect the [HIV] drugs. When they are seriously sick [they] go to the clinic. That is when they accept [it]’.

Peter (27), also from Zambia, noted: ‘a lot of people recognise [us] and when they do, they might be laughing at me. If it is woman, they might think that men won’t be asking them out and if it is a man, women will not be accepting their proposals because they will know he is sick’. The implication is that being identified, or visibly living with HIV, renders a person potentially less desirable to potential intimate partners.

Many participants presented testing with the intervention health workers as a welcome alternative. Joy (36, South Africa, living with HIV), explained her preference for door‐to‐door testing:

People are reluctant to leave houses and go to the clinics for testing, or to go to that particular tent [mobile testing] … They are shy to be seen. People will know that ‘here is so and so, he has entered that tent’ … At the clinics, it’s because of eyes (being seen/watched), so they are wary of people.

In‐home HIV testing, however, meant that HIV became a topic of discussion in intervention households, even if only for the duration of the annual CHiPs’ visits. While the household presented a potentially ‘safe’ environment when compared to public health facilities, the experiences of participants showed that challenges with confidentially and visibility persist. The CHiPs were supposed to find a private space to offer HIV testing in participant homes but in most communities, the physical layout of (often informal) houses with many residents meant that it was difficult to do (Viljoen *et al*. 2020). While privacy could be negotiated, even in crowded spaces, the ability to do so was predicated on relations between household members. Strained relations made it harder to create the desired privacy. Although in‐home testing was for many participants a better alternative than the explicit visibility of public health facilities, for some participants, the door‐to‐door service was described as a threatening experience and as an invasion into the intimate privacy of the home. Brenda (25), a Zambian woman living with HIV, explained that taking an HIV test at home meant that there would be unwanted HIV‐status disclosure:

Your parents, and everyone, or your neighbours will be there. And when they [CHiPs] tell you the results, everyone will get to know [your status]. It is automatic that the people around will be forced to hear those results.

The CHiPs and their services were at times met with distrust. Many participants, especially in South Africa, questioned the confidentiality of the CHiPs and the quality of their services. The familiarity of the CHiPs, as fellow community members, added another layer of complexity. Ramona (28), from South Africa, explained:

Ramona: God, there's one of them wearing that red shirts [referring to a CHiP walking past]. Oh damn. That's a vile woman. She is Paul's sister. She lives up here in the newly built area. She thinks the world of herself.

Researcher: Have you ever tested by them?

Ramona: Yes, the first time. I just want [to say], if we knew you and we are comfortable with you, then we won't allow you to come test us.

The underlying concern Ramona described was related to the expectations that, if she were to test positive for HIV, her status would not be confidential, but would be shared in the community by the CHiPs. Again, the fear of HIV visibility is related to anticipated stigma, or the expected social repercussions of living with HIV, rather than actual experiences of stigma.

Building on counselling approaches in similar sub‐Saharan contexts, the in‐home intervention was explicitly designed to encourage household counselling, couples counselling and disclosure within a household unit. For household members to test together for HIV with the CHiPS, they needed to provide verbal consent. However, the CHiPs’ imposed position in the private sphere of the home meant that, in times of unexpected positive diagnoses, the CHiPs were experienced as unwelcome, or even threatening, despite the process of consent. It was the anticipated consequences of the unwanted disclosure that affected people; potential social shame, blame and concerns over losing standing in the community. Katryn (45), from South Africa, reflected on her experience testing positive for HIV with the CHiPs and the unintended consequences of the interaction:

Researcher: Why did you tell Harris [your son] first?

Katryn: (pause) He heard.

Researcher: They gave your results, in front of your child?

Katryn: Hmm [yes], in front of everyone.

Researcher: That was the first time you knew [about your status]?

Katryn: Yes.

Researcher: How did you feel that they said it in front of everyone?

Katryn: They are outside people ‐ I actually felt a bit hurt.

Researcher: Because you didn’t have control over it?

Katryn: You understand.

The story of the unwanted disclosure of Katryn’s HIV status in front of her children, however, does not end at this point. Despite being initially upset, Katryn explained that the unexpected positive diagnosis and ensuing disclosure meant that she was able to access both physical and emotional support and care from her family. Katryn went on to access HIV treatment outside of national guidelines and regularly went to the clinic to check her health.

The in‐home intervention presented the opportunity to avoid public health facilities and the associated visibility. However, for some, it produced a different type of visibility in the intimate, domestic space and an increase in anticipated stigma. For others, facilitated disclosure through the CHiPs enabled access to social support and care.

### Responsibilisation: Individual blame vs collaborative support

The HPTN 071 (PopART) intervention introduced a dramatic increase in HIV service availability (Hayes *et al*. 2019). The CHiPs were also tasked with distributing the message about the availability of HIV testing and treatment. However, in our discussion with participants, we observed potential subtle narrative shifts. Some participants suggested that, beyond the *availability* of services, community members have the *responsibility* to make use of these now readily available testing or treatment services. Thandi (35, intervention community, South Africa), for instance, told us:

A person is a killer of his/her own self with HIV. HIV doesn’t kill anyone, it kills someone who wants to die. A person would stop taking treatment. A person who stays with [the PLHIV] should then rush them to the clinic… That’s what HIV [positive] people do. That is why prevalence is rising high and not decreasing ‐ they [PLHIV] don’t take the treatment.

Thandi described how PLHIV were not taking responsibility for their own health. People who were not on (supposedly easily accessible) treatment and who did not engage with the ‘logical choice’ (Mol 2008) of accessing care, were not only held responsible, but blamed for their deteriorating health and HIV transmission. These sentiments of ‘taking care of yourself’ (Bond *et al*. 2016) were also echoed in the response from Buhle (65, intervention community, South Africa): ‘Someone who doesn’t take his treatment doesn’t have time for his own life [doesn’t know the value of life]’.

Ben (48), living with HIV in a Zambian intervention community, also described people who did not make use of the HIV services:

Some of them just want to waste their lives, because they have failed to understand what getting tested is all about. They are too ashamed… getting tested does not mean that you are going to die. […] Some people have a low thinking capacity. If a doctor advises you properly on an issue, you have to follow what the doctor tells you.

Shame, or being ashamed, is a key aspect of stigma (Scambler 2006). Ben shamed people who did not test or access ART by describing them as ‘wasting their lives’. Responsibilisation, or the expectation that people should be self‐governing citizens (Foucault 1991) who take care of their own health, was emphasised. PLHIV were not only expected to take on the logical responsibility of taking care of their own health, but also the moral responsibility of curbing transmission (Bond *et al*. 2016). The neoliberal move towards individualism positions those who did not take up personal responsibility for accessing and services related to good health (such as testing and treatment) as social transgressors (Brown and Baker 2012).

Responsibilisation is one way through which blame can be shifted towards those who do not access HIV services (Newman *et al*. 2015). However, we also found that, for some, responsibilisation could be employed to ensure positive health outcomes. Responsibilisation positioned individuals as autonomous empowered citizens and was used by some PLHIV to assert agency in their own health. Several participants related how HIV testing was a method of self‐care. Theo, (intervention community, Zambia), for instance, explained: ‘It is very important for me to test for HIV … I would like to know how to take care of myself based on my status’.

For others, responsibilisation translated as a collaborative, communal effort and ‘the responsibility to take care of each other, for each other, or because of each other’. Ben (48), who was diagnosed with HIV with his wife, explained their decision of initiating HIV treatment:

I convinced my wife that what had befallen us was not good, but since our children are still young, we should go to the clinic … [and] started medication … [We] started asking each other questions as to how we could have contracted the virus, but we realised that the more we asked questions, the more worried sick we became. For the sake of our children … we decided to come to the clinic.

Several community members had either not heard of or did not ‘trust’ the notion of HIV treatment as a form of prevention. Clifford (48), despite articulating how ART works in the body, did not translate this working of ART as a way to prevent transmission:

ART … makes someone regain their strength and then they work in such a way that they make the virus dormant so that the cells can work the way they should. [But] he has a virus. Even if he is on ART, he can still infect another person.

In both Zambia and South Africa, participants were still processing the message of HIV treatment as a form of prevention and most participants were either unfamiliar with or had misconceptions of the prevention capabilities of ART. It is not yet clear how UTT, and the normalising of treatment as a form of HIV prevention might impact HIV stigma in future. From our data, there are indications that responsibilisation for accessing HIV services (including testing and treatment) can lead to shaming, and othering those who are deemed devious – who choose not to access HIV services or adhere to treatment.

## Discussion

By using the concepts of normalisation, HIV visibility and responsibilisation, we have shown how changes to HIV services influenced stigma in complex and nuanced ways. The annual delivery of HIV testing in the private sphere of the home by the CHiPs meant that there was a growing acceptance of HIV‐related services – and that HIV (or testing at least) was normalised, which reduced the stigma associated with testing. However, while HIV testing might have been accepted as the norm, many participants still feared the social repercussions of being diagnosed with HIV. Alongside this, there are a growing set of responsibilities expected of those who test positive, the failure to comply with which can lead to new forms of stigma.

People from all trial communities (intervention and control) reiterated their fears of being identified or suspected of living with HIV when accessing HIV services. For some, concerns of ‘being seen’ were confined to the duration of accessing services at public health facilities. Participants in intervention communities welcomed the new in‐home intervention. This meant that certain HIV services (such as testing), moved away from the public gaze, and into the private sphere of the household. However, participants now became vulnerable to other types of unwarranted disclosure, including to household members. Many participants were wary of the CHiPs, as fellow community members, and their ability to maintain confidentiality. Anticipated stigma remained a concern for people within and outside of the intervention communities. In intervention communities, many participants were able to exchange potential public disclosure at health facilities with the intimacy of in‐home testing and some participants were able to use it as a way to garner support and care for their HIV diagnosis.

As HIV testing became more common, and messaging around testing and treatment increased, new dynamics emerged around responsibilisation. People who did not access the increasingly available services were described as deviant, irresponsible, or careless. Most participants were not aware of how HIV treatment can be employed as a form of prevention. Different forms of responsibilisation might shape stigma once the messaging around treatment as prevention becomes the norm.

As a strength, our data set included detailed, longitudinal, ethnographic data. We were able to compare across conditions (intervention and control), and communities. Through a robust analysis of the large dataset, we were able to show the ways in which stigma is amplified or mitigated in communities. Although we provide a detailed description of the dynamics at play at household level, we have not used quantification of these dynamics to prove statistically significant trends.

Previous research has suggested that increased access to HIV services will lead to a decrease in HIV‐related stigma (Roura *et al*. 2009b). We found that, although community members were more accepting of HIV testing and are, in some cases, better able to navigate disclosure, anticipated stigma and blaming of PLHIV remain pervasive concerns. Some aspects of HIV testing remain exceptional and stigmatised and require specific interventions.

In this manuscript, we have built on the theoretical concepts of normalisation, visibility and responsibilisation as they pertain to HIV stigma. Up to this point, few authors have explored the implications of expanded access to HIV testing in terms of ‘normalisation’. By drawing on empirical data we describe how increased access to testing and the shifting of health services into the private sphere of the home impacts HIV‐related stigma. We found that, despite the acceptance of increased HIV services (and testing specifically) as ‘normal’, HIV normalisation was constrained by other social relationships, rendering the possibility of a diagnosis as potentially life altering. In addition, while some of the concerns of being identified at public facilities were alleviated, the visibility of HIV and a positive diagnosis are subject to different but still challenging conditions, even when delivered at scale and in home.

More telling, the neoliberal positioned expectation of personal responsibility for accessing readily attainable testing options was presented in the narratives of participants in both Zambia and South Africa. These narratives of responsibilisation are potentially born from the historic demand for individual rights, including HIV treatment, in a post‐apartheid South Africa. In Zambia, the emphasis on responsibility stems partly from the religious moral code found in many communities (Bond *et al*. 2016). In both countries, however, participants positioned accessing HIV treatment either as a personal responsibility (as a way of taking care of one’s own health) (Newman *et al*. 2007) and/or as an expectation of others, who, if they refuse to take up the opportunity, risks being shunned by the community.

However, despite these shifts, there are still uncertainties in terms of how universal access to treatment impacts HIV‐related stigma. At this stage, community members in our cohort were unaware, uncertain, or wary of the mechanics of HIV treatment as a form of prevention. To counter anticipated stigma and the responsibilisation of PLHIV, heath implementers need to incorporate messaging focusing on anticipated stigma, countering blame narratives. While this research provides the groundwork for understanding changes in stigma, more research is needed to understand these changes on a granular level as HIV treatment is increasingly becoming the norm.

## Author contribution

**Lario Viljoen:** Conceptualization (lead); data curation (supporting); formal analysis (lead); investigation (lead); methodology (lead); writing‐original draft (lead). **Virginia A. Bond:** Conceptualization (supporting); formal analysis (supporting); funding acquisition (equal); investigation (supporting); methodology (equal); project administration (equal); writing‐review & editing (equal). **Lindsey J. Reynolds:** Conceptualization (supporting); supervision (equal); writing‐review & editing (equal). **Constance Mubekapi‐Musadaidzwa:** Formal analysis (supporting); writing‐review & editing (supporting). **Dzunisani Baloyi:** Data curation (supporting); formal analysis (supporting); writing‐review & editing (supporting). **Rhoda Ndubani:** Data curation (supporting); formal analysis (supporting); methodology (supporting); writing‐review & editing (supporting). **Anne Stangl:** Conceptualization (supporting); funding acquisition (equal); writing‐review & editing (supporting). **Janet Seeley:** Conceptualization (supporting); writing‐review & editing (supporting). **Triantafyllos Pliakas:** Writing‐review & editing (supporting). **Peter Bock:** Funding acquisition (equal); writing‐review & editing (supporting). **Sarah Fidler:** Funding acquisition (equal); writing‐review & editing (supporting). **Richard Hayes:** Funding acquisition (equal); writing‐review & editing (supporting). **Helen Ayles:** Funding acquisition (equal); writing‐review & editing (supporting). **James R. Hargreaves:** Conceptualization (supporting); funding acquisition (equal); project administration (equal); writing‐review & editing (supporting). **Graeme Hoddinott:** Conceptualization (supporting); formal analysis (supporting); investigation (supporting); project administration (supporting); writing‐review & editing (lead).

## Data Availability

The data that support the findings of this study are available from the corresponding author upon reasonable request.

## Note

1 Due to large traveling distances, fieldwork in Zambian were structured around 6‐week ‘blocks’. In South Africa, researchers were located closer to field sites and shorter, more frequent visits were possible. The number of interactions with households varied as researchers were responsive to participant needs, preferences and availability, and the time required for discussions with larger households.
